# Case of Catalepsy, with Remarks

**Published:** 1856-09

**Authors:** H. W. Caffey

**Affiliations:** Collirene, Alabama


### ARTICLE II.

*Case of Catalepsy, with Remarks.* By H. W. CAFFEY, M. D., Collirene, Alabama.

In the course of my practice last year, I met with an interesting and unique case; one which occasioned a great deal of perplexity of mind, as to the proper method of treatment adapted to it, and I am still uncertain whether the measures then instituted were correct, and with the hope of eliciting some light, which may contribute towards dispelling the incertitude by which it was environed, I am induced to forward an account of it. If you deem its merits sufficient to be entitled to a publication, it is at your disposal.

I was sent for in great haste on the 14th of May, 1855, to visit Amanda, a mulatto servant girl, aet. circ. annos, 22, melancholic temperament, and of good general health. On interrogating the messenger, he replied that it was thought she had been bitten by a snake. I arrived at the spot where she lay, about 12 o'clock, M., and on inquiry into the case, was informed that she complained of no indisposition in the morning, was apparently as well as usual, and ate heartily of breakfast; while working in the field about 10 o'clock, A. M., she suddenly fell prostrate on her back; after remaining quiescent a few minutes, she uttered the exclamation, "glory! glory!" and then remained mute and entirely motionless. Some strong linament, composed of camphor and chloroform, had been poured over her face, a portion of which ran into her eyes, but no sensation of pain was evinced; her name had been called loudly in her ears, with an equally futile effect. On examination, I found the pulse regular and natural, about 75 beats per minute; respiration free and gentle as that of a slumbering infant; skin soft and somewhat moist; slight and regular winking, or rather quivering of the eyelids, and general rigidity of the muscular system, (voluntary,) the limbs remaining passively in any position in which they were placed. Her general appearance was that of one in a deep and tranquil slumber. Having just commenced the practice of medicine, and being inexperienced, I did not at once recognise that I had to contend with an affection which had puzzled older and wiser heads than mine. I was sorely perplexed; the case could not be ranked under the head of convulsive diseases; the symptoms were not those of hysteria, epilepsy, or apoplexy: there was no sighing, no violent muscular movements, no stertor; it resembled more closely "coup de soleil," and the attending circumstances, viz: that she was working in the sun, and the weather very warm, would seem in some degree to warrant such a diagnosis, but here the resemblance ceased, and the other symptoms were so widely divergent, as utterly to forbid the idea. Being unable to make out a satisfactory diagnosis, I determined to resort to venesection, to meet the indication of rigidity of the muscular system, and probably some congestive action; she was therefore bled to the amount of about xiv. ℥, and afterwards an emetic was administered without much effect—then gave a cathartic, composed of rhei. et sub. mur. merc. As I was returning home, the idea occurred to my mind, that it must be a case of Catalepsy, from my recollection of its description, and on consulting my books, the impression was confirmed. The symptoms enumerated under that head are as follows: "A sudden suspension of thought, of sensibility and voluntary motion; the patient remaining, during the paroxysm, in the position which she (for it is almost always a female) happened to be at the instant of the attack, or in the position in which she may be placed during its continuance; and all this without any notable affection of the functions of organic life."

15th. Visited her at 10 o'clock, A. M., and found her in much the same condition; pulse somewhat increased in frequency, say 85 to 90 per minute; the purgative given the previous day had produced no motion, nor had the urine been evacuated since I left her. The trance, if I may so designate it, seemed so profound, that I determined to make a trial, to test its reality. Some stinging nettles were procured, and rubbed on her legs, arms and bosom, so as to produce large elevations on the surface, similar to those witnessed in urticaria, but this failed to induce the slightest evidence of pain; she was tickled, pinched, and finally a hot iron was applied, so as to blister, but none of these means resulted in motion, or elicited the faintest token that she was conscious of their use; her pulse was carefully examined during their application, and continued to beat calmly and regularly as before. These measures by some, may be considered harsh and unnecessarily severe, but the circumstances implicated will, I apprehend, extenuate them; her master informed me that she was a "malingerer;" and was in the habit of endeavoring to deceive him, and seemed to apprehend that she was playing a studied part, and in order to satisfy him, as well as solve the doubts in my own mind, they were instituted.

I now ordered an enema of a stimulating and antispasmodic character, which brought away a stool of a copious nature, and healthy appearance; placed a blister on the lumbar region, and ordered a teaspoonful of the following every hour: ℞. G. fœtid., g. aloes, pul. rhei., *aa* ℈j.; spts. lav. comp., ʒj.; et aquæ, ℥ij.; misce. enema to be given in the afternoon, if the mixture does not purge.

16th. Some change in the condition of the patient; pulse 100, and some fulness; less color, and more mobility of the muscular system; the medicine given had not acted on the bowels, so the enema was administered, but brought away no fecal discharge. Blister drew well—ordered it to be transferred to the lower cervical region; continue mixture as before, and enema, if it does not purge.

17th. Much improved; consciousness restored, along with muscular action and sensation; makes complaint of pain in the region of the stomach, and much weakness; pulse 85, and natural in volume. Questioned her in regard to being conscious of her condition while in the trance, and whether she experienced pain from any of the means adopted, to test the completeness of the anæsthesia in her case, and she replied negatively. ℞. Pil., hydrarg., ex. coloc., c. et pulv. rhei. iij., and a teaspoonful of the following mixture every hour: ℞. Spts. lav. c., ʒij., sulph. morph. gr. s.s. et aquæ, ℥jss. misce.

18th. Relapsed into cataleptic state; pulse 90, and somewhat full; not so much rigidity as in the first attack; respiration irregular.

19th. Recovered, and condition much the same as on the 17th.

20th. Did not visit her, but there was a relapse as on the 18th.

21st. Restored to consciousness; pulse 75 and good; action on bowels of a healthy character; complains of hunger.

It will be unnecessary to give a minute detail of the relapses, farther treatment, &c., of this singular case, as it would extend this article far beyond its intended limits: suffice it to say, that relapses continued to take place, from time to time, and some of them lasted as much as seven days, during which time the patient took no nourishment, except an occasional spoonful of water or gruel, which was poured with difficulty down the throat. I endeavored to vary the treatment to meet the different indications as they arose. I consulted the best and most approved authorities accessible to me, but notwithstanding my most strenuous exertions, the disease did not entirely succumb. At one time it was held in abeyance for a month, the patient improved in flesh, color, strength, &c., and I laid the flattering unction to my soul, that victory was about to perch upon my banner, but alas for the vanity of human hopes! it soon resumed, in some measure, its wonted sway. The womb was examined with a speculum, but no evidence of disease detected; and as the catamenial function was regular, and natural in quantity, I could not accuse the uterus as being the peccant organ, at least in this respect. It may be proper to remark, that the treatment consisted in counter irritations, antispasmodics, purgatives and tonics perseveringly employed. Her general health has not suffered much—appetite continued good, and she performs a good deal of labor about the house during the interims of her attacks. She is now wearing a seton in her neck; it remains to be seen whether any benefit will accrue. I would observe here, that a case presenting a good deal of similarity to the foregoing, occurred in the same community, (not under my charge,) which resisted every means instituted for some two or three years, and after becoming utterly wearied and disheartened at the result, all medical treatment was abandoned, and after awhile, the patient spontaneously recovered: this case was also in the person of a negro girl.

*Remarks.*

The above is a "bona fide" instance of Catalepsy, if its description in the books is to be accredited; a disease of such rare occurrence and obscure pathology, that many practitioners of sagacity and experience regarded its existence as altogether apochryphal; among these was the distinguished Cullen, who never "met with any instances of it which were not feigned," and therefore attributed the phenomena exhibited in the so-called affection, to the consumate skill of Charlatans and designing imposters; but that it has a legitimate right to a position among the "ills which flesh is heir to," is now so well attested and sustained by the weight of such indubitable authority, that those who are susceptible to conviction, can no longer look upon it as an affection merely existing in the utopia of the imagination, simply because they have not witnessed it with their own eyes.

Catalepsy and Ecstacy, according to the authority of Dr. Joy, are "mere modifications of the same morbid state," and cites a case given by Sauvages, "in which the first part of the paroxysm consisted in violent declamation and gesticulation, and loud singing, the girl being quite insensible to external objects; whilst in its termination, the fit had all the characters of true Catalepsy."

"Vivid dreams or visions of an extraordinary nature occasionally occur, and are firmly impressed on the memory, so that they can be minutely detailed afterwards." This peculiarity was exemplified in the case under consideration; the girl related to me with a great deal of apparent earnestness, that during her trance, she had visited a magnificent white mansion, inhabited by the most beautiful persons she had ever seen, clothed in white habiliments; and they gave her some of the purest and sweetest water to drink she had ever tasted; that among these personages, she recognized her mistress and several fellow servants, some of whom had been dead several years. This account she reiterated repeatedly, and it seemed to have made a firm impression on her mind.

The peculiar pathological condition which gives rise to this wonderful disease, as I before asserted, is exceedingly obscure and indeterminate; "the younger Pinel has attributed the symptoms to an inflammation of the spinal marrow." But this I apprehend is eminently improbable, for where so sensitive and important a structure is laboring under inflammatory action, its effects would be more clearly and unequivocally manifested, and there would scarcely be so apparent a cessation at times, of all symptoms indicative of diseased action, an absence of all tenderness on pressure, as was exhibited in this case; notwithstanding the high authority from which it emanates, I must therefore reject this explanation. Hoffman entertained the opinion, that it arose from a congelation of the nervous fluid, from the fact, that the cases he met with, transpired during cold weather, and I presume he could offer no more rational solution of a problem, whose mystery puzzled him; but this is evidently an erroneous hypothesis, for the one above related occurred in the summer, while the weather was very warm. The cause assigned by Dr. Joy, I regard as much more philosophical than either of the others, and will more nearly explain the various phenomena displayed, viz: "a temporary state of irritation, and congestion of the cerebro spinal axis." Whether this be the true solution of a difficult problem, time and experience, those great arbiters of human opinions, must determine.

My explanation at the time of the occurrence of the case, was different from any of the above opinions; but after events rendered it very questionable in my mind if I was not in error; however, I looked upon it in this wise: the attack occurred at a catamenial epoch, and her master informed me, that previous to this time, at each monthly period, her mind seemed to be somewhat alienated, evinced by depression of spirits, irritability, and losing its equilibrium from the slightest causes, and then a tendency to run away was developed; indeed, she had left home several times while in this condition, and wandered about like one demented, and when sent home by any of the neighbors whose houses she visited, wept bitterly. She was kindly treated by her master, and could not have acted so from abuse. My explanation ran thus: that there existed sub-acute inflammation of the uterus, and at the transpiration of each monthly flux, a disproportionate degree of congestion took place, then an abnormal degree of irritation being excited, the cerebrum sympathised, through the medium of the ganglionic system of nerves, with the afflicted organ, and exhibited that sympathy through a perturbed or perverted intellection. She is deemed somewhat fatuous at best, and the brain being the weakest point, according to physiological principles, the disease was invited or attracted to that organ. That the brain can sympathise with a disturbed viscus, to such an extent as was exhibited in this case, can be sustained by analogical reasoning. Hypochondriasis is a case strongly in point, the seat of which is undoubtedly in the stomach and bowels; yet the mind is influenced in an extraordinary degree, loses its healthful tone, and is guilty of a thousand fanciful vagaries, existing only in a diseased imagination. Irritation in the bowels of children from worms, acrid ingesta, &c., affecting the brain and spinal marrow, giving rise to convulsions, may also be cited. Irritation in the gums during teething, leading to a like result, is another instance. Many more might be advanced, were it necessary, to substantiate my position, but this is supererogatory, as I believe the fact is not denied.

I had neglected to mention that my patient has never been enciente; whether this would have any influence or not, I am unable to conjecture. As I before intimated, this hypothesis suggested itself to my mind in the inception of the case, as a rational solution of the phenomena displayed, but from the fact that the succeeding attacks came on, irrespective of the catamenial periods, I was led to doubt its correctness.

I submit the case, and my views of it, with much diffidence, with the additional observation, that as this is my primordial effort at writing an article for the consideration of my professional brethren, I trust its want of facts, and imperfections of reasoning, will be regarded with a lenient eye: these are errors to be corrected by sagacious observations and enlarged experience.

